# Institutional delivery in public and private sectors in South Asia: a comparative analysis of prospective data from four demographic surveillance sites

**DOI:** 10.1186/s12884-016-1069-7

**Published:** 2016-09-20

**Authors:** Sushmita Das, Glyn Alcock, Kishwar Azad, Abdul Kuddus, Dharma S. Manandhar, Bhim Prasad Shrestha, Nirmala Nair, Shibanand Rath, Neena Shah More, Naomi Saville, Tanja A. J. Houweling, David Osrin

**Affiliations:** 1 SNEHA (Society for Nutrition, Education and Health Action), 310, Urban Health Centre, 60 Feet Road, Dharavi, Mumbai, 400 017 Maharashtra India; 2 UCL Institute for Global Health, 30 Guilford Street, London, WC1N 1EH UK; 3 Perinatal Care Project, Diabetic Association of Bangladesh, 122 Kazi Nazrul Islam Avenue, Dhaka, 1000 Bangladesh; 4 Mother and Infant Research Activities (MIRA), YB Bhavan, Thapathali, GPO Box 921, Kathmandu, Nepal; 5 Ekjut, Plot 556B, Potka, Chakradharpur, West Singhbhum, Jharkhand India; 6 Department of Public Health, Erasmus MC University Medical Center Rotterdam, P.O. Box 2040, 3000 CA Rotterdam, The Netherlands

**Keywords:** Delivery, Obstetric, Asia, Health facilities, Private sector, Public sector

## Abstract

**Background:**

Maternity care in South Asia is available in both public and private sectors. Using data from demographic surveillance sites in Bangladesh, Nepal and rural and urban India, we aimed to compare institutional delivery rates and public-private share.

**Methods:**

We used records of maternity care collected in socio-economically disadvantaged communities between 2005 and 2011. Institutional delivery was summarized by four potential determinants: household asset index, maternal schooling, maternal age, and parity. We developed logistic regression models for private sector institutional delivery with these as independent covariates.

**Results:**

The data described 52 750 deliveries. Institutional delivery proportion varied and there were differences in public-private split. In Bangladesh and urban India, the proportion of deliveries in the private sector increased with wealth, maternal education, and age. The opposite was observed in rural India and Nepal.

**Conclusions:**

The proportion of institutional delivery increased with economic status and education. The choice of sector is more complex and provision and perceived quality of public sector services is likely to play a role. Choices for safe maternity are influenced by accessibility, quantity and perceived quality of care. Along with data linkage between private and public sectors, increased regulation should be part of the development of the pluralistic healthcare systems that characterize south Asia.

**Electronic supplementary material:**

The online version of this article (doi:10.1186/s12884-016-1069-7) contains supplementary material, which is available to authorized users.

## Background

Skilled birth attendance by a qualified healthcare provider is a critical requirement for safe maternity. Despite progress over the last two decades, inadequate care during pregnancy and delivery is largely responsible for an annual estimated 287 000 maternal and 2.9 million neonatal deaths worldwide [1]. Globally, about two-thirds of births take place in a health facility. However, in sub-Saharan Africa and South Asia - which together contribute over 85 % of maternal deaths - only half of deliveries are institutional [2].

The last two decades have seen considerable increases in institutional delivery rates in South Asia: from 4 to 29 % in Bangladesh between 1993 and 2011, from 26 to 79 % in India between 1992 and 2011, and from 8 to 35 % in Nepal between 1996 and 2011 [3–8]. There are, however, substantial urban-rural disparities in utilization of delivery care services. In most sub-Saharan African and South Asian countries, the proportion of institutional births in urban areas is double that in rural areas (76 %:40 %) [2]. In Bangladesh (2011), 49 % of urban women delivered in a health facility, compared with 23 % of rural women [4]. Comparative proportions were 71 and 31 % in India, and 52 and 15 % in Nepal [9].

National governments have taken measures to encourage institutional delivery and accelerate the slow and uneven improvement in maternal and neonatal health indicators. In 2007, Bangladesh introduced a pilot maternity voucher scheme through which mothers receive a payment for attending antenatal care and delivery at a public or private facility, or at home with a skilled birth attendant [10]. The scheme has reached more than 10 million people (~7 % of the population) across 31 sub-districts [11, 12]. Nepal began a safe delivery incentive scheme in 2005 and free deliveries have been available at government facilities since 2009 through the *Aama Surakshya* programme [13]. The Government of India launched the *Janani Suraksha Yojana* (JSY) - a national conditional cash transfer scheme - in 2005 to incentivize women of lower socioeconomic position to deliver at health facilities [14]. Launched in the same year, the Accredited Social Health Activist (ASHA) program, part of the National Health Mission, promotes institutional delivery for pregnant women in rural areas. ASHAs are trained local female community health volunteers who receive performance-based incentives for identifying pregnant women and helping them access health facilities [14, 15].

A linked approach is to encourage greater private sector participation in healthcare delivery. Concerns about the ability of governments to finance health services adequately, the poor performance of public service delivery systems [16], and the desire to expand client choice have led some countries to encourage the expansion of private sector healthcare [17]. In 2007, the Government of Gujarat, India, launched the *Chiranjeevi Yojana*, a public-private partnership in which the state pays accredited private obstetricians a fee for providing maternity services for poorer women [18, 19]. Some argue that inclusion of the private sector could allow governments to better target the poor and other vulnerable populations [20], and a trend toward increased share in maternal health service provision has been seen over the last decade. In many low-income countries the private sector now manages 40–50 % of health infrastructure and has broad and deep reach [21]. In Bangladesh, private sector institutional delivery care increased from 8 to 17 % between 2007 and 2011, in India from 11 to 21 % between 1993 and 2005, and in Nepal from 4 to 7 % between 2006 and 2011 [4, 8, 9].

Uptake of delivery care services is challenged by a range of factors and identifying and improving them is a priority. The literature suggests that, in addition to availability, distance, cost and quality of services, socio-economic and socio-demographic position are important predictors of institutional delivery [22–28]. Recent studies have highlighted associations with women’s economic status, educational status, age, and parity [29–31]. Our objective in this analysis was to document the share of private maternity care in different settings. Using data collected in community-based studies in Bangladesh, Nepal, and India, we aimed to examine the proportions of institutional deliveries provided by the private sector. We also examined the influence on place of delivery of household economic status, maternal education, age, and parity.

## Methods

### Study populations

We sourced information on maternity care from the surveillance systems of four cluster randomized controlled trials conducted in socio-economically disadvantaged communities between 2005 and 2011. They were run in three underserved rural districts in Bangladesh (Bogra, Maulvibazaar, and Faridpur) [32], in Dhanusha district in the southern Nepal Terai [33], in rural Jharkhand and Odisha states in eastern India [34], and in informal settlements in Mumbai [35]. Table 1 describes the characteristics of each study, including the types of health facilities available.

**Table 1:** Characteristics of studies and populations

Study (Country)	Bangladesh rural [32]	Nepal rural [33]	India rural [34]	India urban [35]
Location	Bogra, Maulvibazaar Faridpur districts	Dhanusha district, southern Nepal	Keonjhar, West Singhbhum, Saraikela districts, Jharkhand and Odisha	Mumbai informal settlements
Period	2005–2011	2008–2011	2005–2008	2006–2009
Estimated population	532 900	240 000	114 000	283 000
Cluster characteristics	Villages making up a union	Village Development Committees	8–10 villages with residents classified as Scheduled Tribe or Other Backward Class	Settlements in 6 municipal wards
Method of cluster identification	Purposive sampling of three districts and clusters within districts	Random selection of 60 clusters from a list of 79	Purposive sampling of three districts and clusters within districts	Random selection of 48 clusters from a list of 92.
Clusters	9	30	18	24
Cluster retention individual retention	All clusters followed up Phase 1 interviews completed after 82 % of identified births Phase 2 after 99 % of identified births	All clusters followed up	All clusters followed up Interviews completed after 98 % of identified births	All clusters followed up Interviews completed after 83 % of identified births
Health facilities available in control areas	Public facilities: District Hospitals, Maternal and Child Welfare Centres, Upazilla Health Complexes. Private facilities: small-to-medium-size clinics, BRAC (NGO) facilities where deliveries do not take place, larger private hospitals with and without comprehensive emergency obstetric care.	Public facilities: five Primary Health Care Centres, ten Health Posts and 88 Sub-Health Posts, none equipped for emergency obstetric care. Refer to public Zonal Tertiary Hospital and private providers for emergency obstetric care.	Public facilities: District Hospitals, Primary Health Centres in which deliveries can notionally take place but that are not usually equipped for comprehensive emergency obstetric care, Community Health Centres acting as referral centres for emergency obstetric care; district hospitals. Private and charitable facilities: medium-sized missionary hospitals with emergency obstetric care.	Public facilities: Municipal tertiary hospitals, general hospitals, maternity homes. Private facilities: specialty hospitals, general hospitals, maternity homes.

### Health system contexts

Bangladesh made steady progress in almost all the health-related Millennium Development Goals (MDGs) over the past decade, through public and private sector activities. It attained the MDG 4 target for reduction in child mortality rate and has a falling maternal mortality ratio (MMR), one of the MDG 5 indicators. To achieve the MDG 5 target of births attended by skilled health personnel, the maternal health voucher scheme was operational in two of the three districts covered by our study (Faridpur and Maulvibazaar). Public facilities in all three districts included district hospitals, maternal and child welfare centres, and *upazilla* health complexes. Private facilities included small-to-medium-size clinics, BRAC (non-government) facilities, and larger private hospitals.

Nepal attained the MDG 4 target for reduction in child mortality and marginally attained the MDG 5 target for reduction in MMR. There has been some consideration of how this was achieved, given a widespread breakdown in governance during the insurrection, but a national Maternity Incentive Scheme (MIS) was implemented in 2005 and was operational during data collection. Dhanusha district had one zonal hospital, a private medical college hospital equipped for comprehensive obstetric care, public health posts, and a range of small private facilities. At the time of data collection the MIS was available only through public health providers.

India almost attained the MDG 4 and MDG 5 targets. The rural data from India were collected between 2005 and 2008. The JSY was launched in 2005 and beginning to be operational in study areas towards the end of 2008, so that its impact was unlikely to be reflected. In the urban setting, mothers with Below Poverty Line cards were eligible for the JSY cash incentive for delivery at a health facility [36]. The urban India study included informal settlements in Mumbai with a wealth of public and private providers [37]. Public facilities included primary, secondary, and tertiary institutions run by the municipal corporation. Municipal and government health infrastructure together constituted half of the inpatient care available in the city, the rest being provided by private hospitals and clinics.

### Data collection

The participants were women of reproductive age (15–49 years) who delivered in the study areas during the data collection period and who consented to be interviewed around six weeks after delivery. All the sites operated vital registration systems that monitored births, stillbirths, and neonatal deaths identified by female community-based informants covering 250–350 households each. Reports were verified by trained interviewers and informants remunerated for correct identification. In Bangladesh, rural India, and urban India, women were visited six weeks after delivery for a postpartum interview. In Nepal, births in the study area were registered and interviews conducted after all births in small clusters and in a random sample of 10 births per month in larger clusters. Interviews were predominantly based on closed questions about antenatal, delivery, and postnatal events.

### Dependent variables

The outcome of interest was institutional delivery in the public or private sector, based on the reported type or name of the institution.

### Independent variables

We chose variables purposively from the available dataset, to reflect socioeconomic position (household asset index, maternal schooling) and demography (maternal age, parity). Household economic position was defined by tertiles of an asset index developed from standardized weights of the first component of a principal components analysis [38, 39]. Scores were calculated separately for each site. Maternal education was categorized as none, primary, secondary, or higher. Maternal age was categorized as under 20, 20–24, 25–29, or 30+ years. Gravidity was represented by a binary variable: primigravid or multigravid.

### Statistical analysis

The original trials were designed to evaluate the impact of participatory women’s groups on maternal and neonatal health outcomes [32–35, 40], and we restricted our analysis to residents of control areas who reported a birth in the trial period. We summarized sites of delivery and choice of provider by socio-economic and socio-demographic position, with frequencies and percentages. The denominators were all deliveries for institutional delivery, and institutional deliveries for private sector delivery. We entered private sector institutional delivery as the dependent variable in a series of univariable logistic regression models, including a random effect for cluster, for each of the four independent variables. A multivariable model included all four of them as covariates. The analysis was run for each trial separately in Stata13 (Stata, College Station, TX, USA).

### Ethical approval

Data for the study originated with trials that had received ethical approval from the ethics committee of the Diabetic Association of Bangladesh, an independent ethics committee in Jamshedpur, Jharkhand, India, the Nepal Health Research Council, the Municipal Corporation of Greater Mumbai and the Independent Ethics Committee for Research on Human Subjects (Mumbai, India), and the ethics committee of the Institute of Child Health, University College London.

## Results

The data described 52 750 deliveries across four sites: 23 608 in rural Bangladesh, 14 079 in rural Nepal, 8978 in rural India, and 6085 in informal slum settlements in urban India. Table 2 summarises characteristics of the participants. In rural Nepal and India, most women had not been to school. Generally, when women had done so they had attended up to secondary level at all sites. Adolescent pregnancy appeared more common in rural Nepal, and women had their pregnancies later in urban India. About one-third of women were delivering their first child.

**Table 2:** Participant characteristics

	Bangladesh rural 1	Bangladesh rural 2	Nepal rural	India rural	India urban
	*n* (%)	*n* (%)	*n* (%)	*n* (%)	*n* (%)
Maternal schooling					
No schooling	4210 (28)	1565 (18)	10365 (74)	6084 (68)	1708 (28)
Primary	5311 (36)	2754 (32)	1634 (12)	464 (5)	315 (5)
Secondary	5327 (36)	4176 (48)	1970 (14)	2351 (26)	3511 (58)
Higher	99 (<1)	117 (1)	30 (<1)	79 (1)	551 (9)
Missing	-	49 (1)	80 (<1)	-	-
Maternal age (y)					
< 20	2285 (15)	1314 (15)	2872 (21)	1116 (12)	496 (8)
20–24	5685 (38)	3374 (39)	5526 (39)	2907 (32)	2576 (42)
25–29	3974 (27)	2342 (27)	3920 (28)	2420 (27)	2068 (34)
30–34	1911 (13)	1118 (13)	1159 (8)	1297 (15)	710 (12)
35+	1087 (7)	513 (6)	601 (4)	707 (8)	226 (4)
Missing	5 (<1)	-	1 (<1)	531 (6)	9 (<1)
Gravidity					
First pregnancy	5086 (34)	2911 (34)	4582 (33)	2520 (28)	1795 (29)
Not first pregnancy	9861 (66)	5747 (66)	9497 (67)	6457 (72)	4290 (71)
Missing	-	3 (<1)	-	1 (<1)	-
Asset index					
Poorest	6373 (43)	3478 (40)	4713 (33)	5319 (59)	3194 (53)
Poor	3658 (24)	2400 (28)	4720 (34)	2159 (24)	937 (15)
Least poor	4916 (33)	2783 (32)	4645 (33)	1500 (17)	1954 (32)
Missing	-	-	1 (<1)	-	-
Total	14947 (100)	8661 (100)	14079 (100)	8978 (100)	6085 (100)

Table 3 summarizes the proportions of deliveries that were home or institutional, and, when institutional, public or private. There was variation between sites in the proportion of institutional delivery, which was routine in urban informal settlements (92 %), but used by less than 30 % of women at other sites. In the event of institutional delivery, there were substantial differences in public-private split, which was 84:16 in rural Nepal, but 23:77 in rural India. The two phases of the rural Bangladesh dataset showed obvious changes over time: the first was collected from 2005 to 2007 and the second from 2009 to 2011. Institutional deliveries were uncommon in the first phase (17 %) and more than half (55 %) of them took place in public facilities. In the second phase, institutional delivery had risen to 29 % and now favored the private sector (58 %). Institutional delivery rates were low in rural Nepal (25 %). The type of institution was not known in 124 cases, but fell predominantly to the public sector (84 %). Institutional delivery was uncommon in the dataset from rural India. When it was chosen, the private sector was preferred in 77 % of cases. Preference for the private sector was much greater in rural than in urban India (42 %). The results for women in informal settlements in urban India included only those who delivered in Mumbai, and not those who went back to their places of origin for delivery (1387).

**Table 3:** Deliveries at four sites, by location

Trial site	All deliveries	Home deliveries	Institutional deliveries	Public sector deliveries	Private sector deliveries
	*N* (%)	*n* (%)	*n* (%)	*n* (% institutional)	*n* (% institutional)
Bangladesh rural 1	14947 (100)	12349 (83)	2598 (17)	1419 (55)	1179 (45)
Bangladesh rural 2	8661 (100)	6181 (71)	2480 (29)	1035 (42)	1445 (58)
Nepal rural	14079 (100)	10543 (75)	3536 (25)	2878 (84)^a^	534 (16)^a^
India rural	8978 (100)	7147 (80)	1831 (20)	426 (23)	1405 (77)
India urban	6085 (100)	499 (8)	5586 (92)	3243 (58)	2343 (42)
Total	52750 (100)	36719 (70)	16031 (30)	9001 (57)	6906 (43)

^a^Institution unclassified for 124 deliveries

**Fig. 1:**
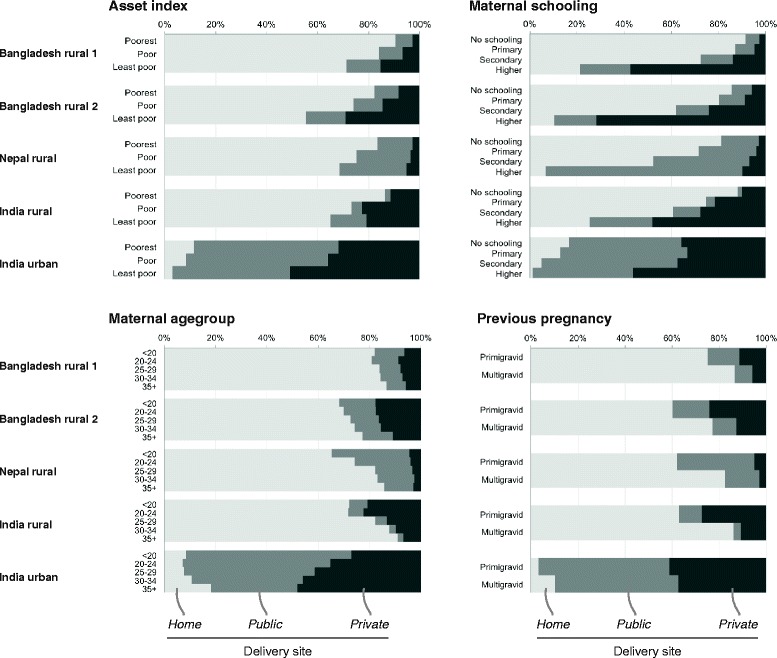
Place of delivery, by economic position, maternal schooling, maternal age-group and gravidity, for four sites

Figure 1 summarizes type of delivery care provider by socio-economic and socio-demographic position (Additional file 1: Table S1). The proportion of institutional delivery increased with economic status and higher educational attainment across all sites. Younger women were more likely to deliver at an institution, although teenagers were slightly less likely to do so than women in their early twenties in rural India and Bangladesh in the earlier phase. When deliveries were institutional, the proportion of private sector use increased with wealth and education, although the difference in urban India was only marked for women with higher education. Greater proportions of younger women delivered in the private sector at rural sites, but private sector delivery increased with age in urban India. Greater proportions of women in their first pregnancies delivered in the private sector, although the difference was small in urban India.

Table 4 summarizes the findings of logistic regression models with private institutional delivery as the dependent variable. Public-private split differed across the sites. Increasing economic status was associated with greater odds of private sector delivery in rural Bangladesh and urban India, but there was a suggestion that poorer women had greater odds of private sector delivery in rural Nepal and India. Increasing maternal education was associated with greater odds of private sector delivery in rural Bangladesh and in the highly educated in urban India, but lesser odds in rural India. The association of increasing maternal age with increasing odds of private sector delivery was confirmed in rural Bangladesh (phase 1) and urban India, but not in rural Nepal and India (although the numbers were smaller). Primigravid women had greater odds of private institutional delivery in rural Bangladesh and urban India, but lesser odds in rural Nepal and rural India.

**Table 4:** Odds ratios for private sector (compared with public sector) institutional delivery at four sites, by asset index, maternal education, age, and gravidity

	Bangladesh rural 1		Bangladesh rural 2		Nepal rural		India rural		India urban	
	OR (95 % CI)	aOR (95 % CI)	OR (95 % CI)	aOR (95 % CI)	OR (95 % CI)	aOR (95 % CI)	OR (95 % CI)	aOR (95 % CI)	OR (95 % CI)	aOR (95 % CI)
Asset index										
Poorest	1	1	1	1	1	1	1	1	1	1
Poor	1.69 (1.32, 2.17)	1.51 (1.17, 1.95)	1.54 (1.22, 1.95)	1.36 (1.07, 1.74)	0.72 (0.55, 0.94)	0.73 (0.56, 0.96)	0.71 (0.49, 1.03)	0.81 (0.55, 1.20)	1.17 (0.99, 1.38)	1.21 (1.02, 1.43)
Least poor	2.87 (2.33, 3.55)	2.16 (1.72, 2.72)	2.35 (1.91, 2.90)	1.71 (1.37, 2.13)	0.80 (0.62, 1.04)	0.84 (0.64, 1.10)	0.50 (0.36, 0.70)	0.71 (0.49, 1.02)	2.03 (1.78, 2.33)	2.02 (1.76, 2.33)
Maternal education										
No schooling	1	1	1	1	1	1	1	1	1	1
Primary	1.31 (0.99, 1.74)	1.17 (0.87, 1.57)	1.18 (0.84, 1.64)	1.12 (0.80, 1.59)	0.88 (0.65, 1.18)	0.90 (0.67, 1.22)	1.06 (0.55, 2.05)	1.15 (0.58, 2.27)	0.82 (0.61, 1.08)	0.84 (0.63, 1.12)
Secondary	2.52 (1.96, 3.26)	1.96 (1.47, 2.62)	2.96 (2.18, 4.01)	2.46 (1.77, 3.41)	0.85 (0.68, 1.07)	0.90 (0.71, 1.14)	0.47 (0.34, 0.65)	0.49 (0.34, 0.70)	0.84 (0.73, 0.97)	0.81 (0.70, 0.94)
Higher	7.44 (4.23, 13.11)	4.39 (2.44, 7.90)	7.95 (4.5, 14.06)	5.47 (3.03, 9.90)	0.32 (0.07, 1.39)	0.33 (0.07, 1.47)	0.21 (0.10, 0.42)	0.27 (0.12, 0.57)	1.55 (1.25, 1.93)	1.25 (0.99, 1.57)
Maternal age										
< 20	1	1	1	1	1	1	1	1	1	1
20–24	1.45 (1.14, 1.84)	1.37 (1.07, 1.77)	1.13 (0.89, 1.43)	1.18 (0.91, 1.53)	1.23 (0.97, 1.57)	1.10 (0.84, 1.44)	1.07 (0.72, 1.58)	1.06 (0.70, 1.60)	1.25 (1.00, 1.58)	1.33 (1.05, 1.69)
25–29	1.60 (1.23, 2.08)	1.81 (1.32, 2.47)	1.16 (0.90, 1.51)	1.41 (1.02, 1.95)	1.46 (1.11, 1.93)	1.17 (0.83, 1.66)	0.81 (0.53, 1.24)	0.67 (0.41, 1.12)	1.55 (1.23, 1.96)	1.75 (1.35, 2.25)
30–34	1.40 (1.02, 1.91)	1.76 (1.21, 2.56)	1.16 (0.84, 1.59)	1.43 (0.97, 2.11)	1.07 (0.68, 1.68)	0.79 (0.47, 1.30)	0.88 (0.49, 1.57)	0.63 (0.32, 1.23)	2.08 (1.59, 2.73)	2.40 (1.78, 3.22)
35+	1.16 (0.78, 1.72)	1.74 (1.10, 2.75)	0.69 (0.45, 1.06)	1.20 (0.73, 1.97)	1.74 (0.98, 3.11)	1.32 (0.71, 2.46)	0.93 (0.41, 2.11)	0.55 (0.22, 1.33)	2.69 (1.84, 3.94)	3.10 (2.08, 4.64)
Gravidity										
Primigravid	1	1	1	1	1	1	1	1	1	1
Multigravid	0.90 (0.77, 1.05)	0.83 (0.67, 1.02)	0.78 (0.66, 0.93)	0.77 (0.62, 0.97)	1.42 (1.17, 1.73)	1.32 (1.03, 1.70)	1.17 (0.89, 1.55)	1.31 (0.92, 1.87)	0.93 (0.82, 1.05)	0.78 (0.68, 0.90)

*OR* odds ratio, *aOR* adusted odds ratio, *CI* confidence interval

Covariates included in adjusted models: asset index, maternal education, age, gravidity

## Discussion

### Findings

Our analysis of data from 52 750 births in three South Asian countries over six years showed broad variation in rates of institutional delivery and public-private split across study sites and between socio-economic and socio-demographic groups. There were substantial differences in institutional delivery proportion between rural and urban areas, by a factor of about three. There were also socio-economic and socio-demographic disparities in institutional maternity care, with women from lower socio-economic and educational backgrounds less likely to have institutional delivery. Use of the private sector differed between sites. In Bangladesh and urban India, it was greater for wealthier, better educated, and older women. The opposite was observed in rural India and, perhaps, in rural Nepal (the numbers were small), in which greater proportions of poorer, less educated, multigravid women delivered at private rather than public facilities.

### Limitations

Our analysis had some limitations. All the participants could be classified as poor according to international metrics. We used principal components analysis to generate asset indices for each site, but the index distributions were highly positively skewed to a degree to which normalization by transformation was not possible. For this reason, quantiles of asset index did not yield groups of equal size from some of the sites. Secondly, classification of institutions as public or private was not possible using the raw data in <1 % of cases. We should also be aware of the variable nature of private care, particularly in terms of its categorization as for-profit or not-for-profit. Institutions such as charitable trusts and non-government organisations were classified as private when in some cases – Bangladesh being a good example – the cost to clients may have been similar to the costs of public sector care. Finally, there have been increases in institutional delivery since the study period. More recent data from the rural India site, for example, suggest that the proportion had reached 50 % by 2011 [41].

### Institutional delivery

The finding of low prevalence of institutional delivery in South Asian countries is consistent with previous research. At three of the four sites, less than one-third of births were institutional. In rural Bangladesh, institutional delivery rates in the first and second phases were low and corroborate national Demographic and Health Survey (DHS) data for the corresponding periods (11 % in 2007 and 23 % in 2011) [4, 42]. In Nepal, home birth continues to predominate, especially in rural areas, and our findings validate the low proportion of institutional delivery reported by the Ministry of Health and Population (25 %) [8]. Neither 24-h comprehensive obstetric care nor the Maternity Incentive Scheme were available at private health facilities in the Nepal site at the time of the study, which may explain the lower usage in this area. In rural India, one-in-five births was institutional, compared with a District Level Household and Facility Survey (DLHS) national figure of 38 % in 2007–8 [43]. This difference is probably due to the fact that the Jharkhand and Odisha sites were home to a high proportion of underserved tribal communities with poor access to health services. In contrast, residents of urban India have access to a range of health services in both public and private sectors. Most of the births in urban Mumbai were institutional (92 %), and this agrees with roughly concomitant data from the third National Family Health Survey (83 %) [44]. The urban-rural differences were somewhat predictable. A systematic review of determinants of maternal health service use found that urban, wealthier women were more likely to have institutional deliveries [45]. Explanations include the fact that women in urban areas tend to be better educated, live nearer to health services, have access to more extensive transport systems, and may have greater autonomy and access to information [46, 47].

### Determinants

Our findings support those of studies that show economic conditions to be an important predictor of institutional delivery [48, 49]. Women of low economic position are less likely to use maternal health services, and we observed a systematic increase in institutional delivery with increasing wealth at all sites. Our analysis also corroborates the findings of studies which suggest that maternal education [50], household wealth, and urban-rural residence have important and consistent effects on utilization of health services for delivery [23, 28, 51–53]. Education is a key determinant of access and utilization. Fewer women had schooling in rural areas of Nepal (26 %) and India (32 %) and we saw fewer institutional deliveries in these areas. In comparison, a higher proportion of women reported institutional delivery in Bangladesh, where educational attainment was higher (72 % of women reported some schooling in phase 1 and 82 % in phase 2). The proportion of institutional delivery was also higher in urban India, where 56 % of women reported completing secondary education. Other determinants of healthcare uptake include age and parity. Most of our study participants were young and one-third were primigravid. Several South Asian studies have shown that women in their first pregnancies are more likely to deliver at a health facility and with a skilled birth attendant [8, 23, 27, 54]. Our findings suggested a substantial influence of parity on utilization of delivery care, with younger women more likely to have their first birth in a health facility.

### Uptake of private sector delivery

Private sector delivery is increasing over time, with positive associations with wealth and education, previous private sector delivery, and – to a lesser extent – primiparity [55]. In rural Nepal, Bangladesh, and urban India, the public sector was still a major provider of care for women who chose institutional delivery. Availability of better public health infrastructure combined with accessibility may have led to increased utilization compared with the private sector in urban informal settlements in India. A shift from public sector to private sector delivery was seen in the second phase of the Bangladesh study.

Private sector healthcare is heterogeneous in the South Asian context of medical pluralism. Bangladesh is served by a mix of public and private sector providers in which not-for-profit facilities play a major part. India, by contrast, has a dominant private sector. Around 70 % of total health expenditure in 2012 was private, 61 % was out-of-pocket, and 75 % of outpatient visits and 62 % of inpatient episodes were in the private sector. Out-of-pocket expenditure at public sector facilities was low, at 2 % [56]. Nepal can be classified as having a “stratified private sector shaped by low incomes and public sector characteristics” [57]. Around 61 % of total health expenditure in 2012 was private, 49 % was out-of-pocket, and 65 % of outpatient visits and 46 % of inpatient episodes were in the private sector. Out-of-pocket expenditure at public sector facilities was 7 % [57].

Bangladesh has seen a steady increase in institutional deliveries [50]. DHS analyses suggest a rise from 2 % in 1996 to 17 % in 2011 [20], most of which was accounted for by use of the private sector [55]. Wealth-related inequality in institutional delivery seems to have fallen [20, 50], more rapidly in urban than in rural areas, and the private sector is becoming the major provider of maternity care [58]. Much of this private provision is not-for-profit [59], and the nongovernmental health sector is now double the size of the government sector [60]. Similar trends were observed in rural India, where more than three-quarters of institutional deliveries were in private facilities. DHS estimates suggest an increase in institutional delivery of about 7 % between 1998 and 2005, most of which was accounted for by the private sector [55]. In this case, private care is predominantly for-profit. Unlike in Bangladesh and India, most of the increase in institutional deliveries in Nepal was accounted for by the public sector [55], although private delivery increased from 1 % in 1996 to 10 % in 2011 [20].

### Preference for private healthcare

Healthcare quality can be seen as a combination of service quality (the user experience) and technical quality (the clinical process). Service quality appears to be higher in the private sector [61–64], and users generally consider private care to be better than public in terms of responsiveness, longer opening hours, appointment systems, shorter waiting times, confidentiality, cleanliness, and privacy [65, 66]. These qualities may appeal to those who can afford them because they respond to women’s busy schedules and desire for positive experiences of health care [67]. The technical quality of private sector services may not be as good as in the public sector [63, 64], but this does not apply across the board: mapping of health facility signal functions across Bangladesh in 2007–2008 suggested that emergency obstetric services such as caesarean section and blood transfusion were available at a higher proportion of private than of public sector health facilities [68], and recent considerations have emphasized the benefits of pluralism [60]. In rural India, and possibly in rural Nepal, concerns about the availability and quality of care may have exacerbated other geographic, financial and social barriers, deterring women from delivering in health facilities unless they experienced antepartum or intrapartum complications [69, 70]. Comprehensive obstetric care was available in the Nepal site only at the regional public hospital and one or two private facilities. If institutional deliveries were often a response to anticipated or actual complications, women may have preferred the private sector if they thought that skilled care, and particularly emergency obstetric care, would be more readily available there.

Economic status is believed to be the strongest influence on choosing between a private-for-profit or public facility for institutional delivery [71]. In Bangladesh and urban India, women from wealthier backgrounds were more likely to deliver at private facilities [72]. In rural Nepal and India, amongst the subset of the population that delivered at institutions that were less poor, better educated, more likely to be younger and primigravida than those delivering at home, economic status was negatively associated with private care. Wealthier women in rural India may have had access to better quality public services, or have been more empowered to navigate their way through the public system. It is also possible that poorer women and those from tribal and Scheduled Caste communities may have feared discrimination in public facilities and avoided them as a result [62, 73–75], despite the burden of out-of-pocket expenditure on private care [76]. Incentive programs have increased institutional delivery in the public sector, but there is a pressing need to monitor quality of care [16]. A recent evaluation of the JSY suggests that the scheme has led to an increase in births in public facilities and substitution away from the private sector. Importantly, poorer women were more likely than wealthier women to give birth in a public health facility in response to the scheme [77], although it did not compensate fully for the financial stress of complicated deliveries [78]. In rural Nepal, the Maternity Incentive Scheme was introduced around the beginning of our data collection and at that time less poor and more educated women were more likely to access it [79]. Since the incentive was only available through public providers, it may have been that over most of the data collection period better-educated and less poor women came to know of it and responded to it by choosing public sector institutional delivery, whilst poorer families were less aware of the scheme and sought private care when they perceived deliveries to be high-risk.

## Conclusions

Although some suggest that the gap between health infrastructure and attainment would best be filled through public-private partnership [80], the evidence for the potential of large, private for-profit services to provide healthcare at scale for low-income groups in low- and middle-income countries is limited and mostly includes providers in urban and peri-urban India [66]. As economic wherewithal and education increase, families move toward institutional delivery. The choice of sector is more complex and the provision and perceived quality of public sector services is likely to play a role, as are institutional delivery incentive schemes. Where a mix of public and private services is available, families arrange for young women to deliver in either. We think that the precise split depends on the reputation of public sector services: fairly good in Mumbai (58 % of institutional deliveries) and less good than private sector services in rural Bangladesh (42 %). This proposition holds for rural Nepal and rural India too, but is augmented by limitations to available services. In rural Nepal, the small proportion of women who chose institutional delivery did so at public sector facilities, which may be less about reputation than about the availability and affordability of private sector delivery in rural areas. Choices for safe maternity are influenced by accessibility, quantity, and perceived quality of care, and the balance between public and private provision shifts with time and place. We need more information on healthcare uptake and activities, including linkage of records from private for-profit and not-for-profit services [60]. We recommend, as have others [59], the inclusion of more refined descriptors of place of institutional delivery in national and subnational surveys.

We support calls for increases in the numbers and training of skilled human resources in the public sector [68]. There is also some evidence that disrespect and abuse associated with maternity services can be reduced through interventions that work with policymakers, train providers, and strengthen links between facility and community [81].

The evidence base for interventions to improve private sector quality in low- and middle-income countries is limited [59]. In a recent review, Montagu and Goodman discuss four approaches: prohibition of private practice, encouragement and subsidy for delivery of selected services, purchase of private services by the public sector, and operational constraint through regulation. In the context of fairly open economies, the first of these - prohibition - has had only limited success. Encouragement of institutional delivery is best represented by the incentive schemes already operational in all three countries, and purchase is best represented by systems like the Chiranjeevi Yojana or by voucher programs [82, 83].

We support calls for regulation, accreditation, and accountability frameworks [60, 64, 84]. Constraint through statutory regulation such as licensing, quality control, or prescription limitations continues to be problematic. There are also some obstacles to self-regulation. Regulation by professional organisations has to take account of the fact that practitioners may work in both private and public sectors, and voluntary accreditation has cost implications and relies on the involvement of a majority of providers. Nevertheless, increased regulation – along with data linkage – should be part of the rational development of the pluralistic healthcare systems that characterize south Asia.

## Additional file

Additional file 1: Table S1. Type of delivery care by socio-economic and socio-demographic position. (DOCX 44 kb)

## Abbreviations

aOR: Adjusted odds ratio
ASHA: Accredited Social Health Activist. India National Health Mission
BRAC: Bangladesh Rural Advancement Committee (Building Resources Across Communities)
CI: Confidence interval
DHS: Demographic and Health Survey
DLHS: District Level Household and Facility Survey, India
JSY: Janani Suraksha Yojana. India safe delivery incentive program
MDG: Millennium Development Goal
MIRA: Mother and Infant Research Activities, Nepal
MMR: Maternal mortality ratio
OR: Odds ratio
SNEHA: Society for Nutrition, Education and Health Action, Mumbai, India
UCL: University College London
