# Supplementary material for: Institutional delivery in public and private sectors in South Asia: a comparative analysis of prospective data from four demographic surveillance sites
Source: BMC Pregnancy Childbirth. 2016 Sep 20;16:273. doi: 10.1186/s12884-016-1069-7 (PMC5029035; doi:10.1186/s12884-016-1069-7)
Supplement: Additional file 1: Table S1. — Type of delivery care by socio-economic and socio-demographic position. (DOCX 44 kb) [file 12884_2016_1069_MOESM1_ESM.docx]

# Additional file 1: Table S1. Type of delivery care by socio-economic and socio-demographic position

|  | **Bangladesh rural 1** | | | | **Bangladesh rural 2** | | | | **Nepal rural** | | | |
| --- | --- | --- | --- | --- | --- | --- | --- | --- | --- | --- | --- | --- |
|  | Home delivery | Public hospital | Private hospital | All | Home delivery | Public hospital | Private hospital | All | Home delivery | Public hospital | Private hospital | All |
|  | n (%) | n (%) | n (%) | N (%) | n (%) | n (%) | n (%) | N (%) | n (%) | n (%) | n (%) | N (%) |
| **Asset index** |  |  |  |  |  |  |  |  |  |  |  |  |
| Poorest | 5772 (91) | 422 (7) | 179 (3) | 6373 (100) | 2862 (82) | 328 (10) | 288 (8) | 3478 (100) | 3931 (83) | 651 (14) | 131 (3) | 4713 (100) |
| Poor | 3075 (84) | 334 (9) | 249 (7) | 3658 (100) | 1779 (74) | 272 (11) | 349 (15) | 2400 (100) | 3550 (75) | 1001 (21) | 169 (4) | 4720 (100) |
| Least poor | 3502 (71) | 663 (14) | 751 (15) | 4916 (100) | 1540 (55) | 435 (16) | 808 (29) | 2783 (100) | 3185 (69) | 1226 (26) | 234 (5) | 4645 (100) |
| **Maternal education** |  |  |  |  |  |  |  |  |  |  |  |  |
| No schooling | 3849 (91) | 248 (6) | 113 (3) | 4210 (100) | 1339 (86) | 133 (8) | 93 (6) | 1565 (100) | 8410 (81) | 1635 (16) | 320 (3) | 10365 (100) |
| Primary | 4624 (87) | 426 (8) | 261 (5) | 5311 (100) | 2204 (80) | 303 (11) | 247 (9) | 2754 (100) | 1171 (72) | 395 (24) | 68 (4) | 1634 (100) |
| Secondary | 3855 (72) | 724 (14) | 748 (14) | 5372 (100) | 2589 (62) | 571 (14) | 1016 (24) | 4176 (100) | 1031 (52) | 802 (41) | 137 (7) | 1970 (100) |
| Higher | 21 (21) | 21 (21) | 57 (58) | 99 (100) | 12 (10) | 21 (18) | 84 (72) | 117 (100) | 2 (7) | 25 (83) | 3 (10) | 30 (100) |
| **Maternal age (y)** |  |  |  |  |  |  |  |  |  |  |  |  |
| <20 | 1874 (82) | 259 (11) | 152 (7) | 2285 (100) | 895 (68) | 185 (14) | 234 (18) | 1314 (100) | 1875 (65) | 867 (30) | 130 (5) | 2872 (100) |
| 20-24 | 4588 (81) | 591 (10) | 506 (9) | 5685 (100) | 2361 (70) | 418 (12) | 595 (18) | 3374 (100) | 4097 (74) | 1202 (22) | 227 (4) | 5526 (100) |
| 25-29 | 3334 (84) | 323 (8) | 317 (8) | 3974 (100) | 1700 (73) | 257 (11) | 385 (16) | 2342 (100) | 3217 (82) | 573 (15) | 130 (3) | 3920 (100) |
| 30-34 | 1609 (84) | 164 (9) | 138 (7) | 1911 (100) | 829 (74) | 115 (10) | 174 (16) | 1118 (100) | 962 (83) | 168 (15) | 29 (2) | 1159 (100) |
| 35+ | 941 (86) | 81 (8) | 65 (6) | 1087 (100) | 396 (77) | 60 (12) | 57 (11) | 513 (100) | 515 (86) | 68 (11) | 18 (3) | 601 (100) |
| Missing | 3 (60) | 1 (20) | 1 (20) | 5 (100) |  |  |  |  |  |  | 1 (100) | 1 (100) |
| **Gravidity** |  |  |  |  |  |  |  |  |  |  |  |  |
| First pregnancy | 3814 (75) | 681 (13) | 591 (12) | 5086 (100) | 1748 (60) | 457 (16) | 706 (24) | 2911 (100) | 2840 (62) | 1506 (33) | 236 (5) | 4582 (100) |
| Not first pregnancy | 8535 (86) | 738 (8) | 588 (6) | 9861 (100) | 4431 (77) | 578 (10) | 738 (13) | 5747 (100) | 7827 (82) | 1372 (15) | 298 (3) | 9497 (100) |
| Missing |  |  |  |  | 2 (67) |  | 1 (33) | 3 (100) |  |  |  |  |
|  |  |  |  |  |  |  |  |  |  |  |  |  |
|  | **India rural** | | | | **India urban** | | | |  |  |  |  |
| **Asset index** |  |  |  |  |  |  |  |  |  |  |  |  |
| Poorest | 4589 (86) | 124 (2) | 606 (11) | 5319 (100) | 360 (11) | 1818 (57) | 1016 (32) | 3194 (100) |  |  |  |  |
| Poor | 1582 (73) | 91 (4) | 486 (23) | 2159 (100) | 79 (8) | 522 (56) | 336 (36) | 937 (100) |  |  |  |  |
| Least poor | 976 (65) | 211 (14) | 313 (21) | 1500 (100) | 60 (3) | 903 (46) | 991 (51) | 1954 (100) |  |  |  |  |
| **Maternal education** |  |  |  |  |  |  |  |  |  |  |  |  |
| No schooling | 5355 (88) | 115 (2) | 614 (10) | 6084 (100) | 281 (16) | 816 (48) | 611 (36) | 1708 (100) |  |  |  |  |
| Primary | 346 (75) | 18 (4) | 100 (21) | 464 (100) | 40 (13) | 170 (54) | 105 (33) | 315 (100) |  |  |  |  |
| Secondary | 1426 (61) | 272 (12) | 653 (28) | 2351 (100) | 171 (5) | 2023 (58) | 1317 (37) | 3511 (100) |  |  |  |  |
| Higher | 20 (25) | 21 (27) | 38 (48) | 79 (100) | 7 (1) | 234 (43) | 310 (56) | 551 (100) |  |  |  |  |
| **Maternal age (y)** |  |  |  |  |  |  |  |  |  |  |  |  |
| <20 | 804 (72) | 79 (7) | 233 (21) | 1116 (100) | 42 (9) | 319 (64) | 135 (27) | 496 (100) |  |  |  |  |
| 20-24 | 2080 (72) | 178 (6) | 649 (22) | 2907 (100) | 182 (7) | 1484 (58) | 910 (35) | 2576 (100) |  |  |  |  |
| 25-29 | 1986 (82) | 111 (5) | 323 (13) | 2420 (100) | 159 (8) | 1052 (51) | 857 (41) | 2068 (100) |  |  |  |  |
| 30-34 | 1136 (88) | 33 (2) | 128 (10) | 1297 (100) | 75 (11) | 308 (43) | 327 (46) | 710 (100) |  |  |  |  |
| 35+ | 643 (91) | 14 (2) | 50 (7) | 707 (100) | 41 (18) | 76 (34) | 109 (48) | 226 (100) |  |  |  |  |
| Missing | 498 (94) | 11 (2) | 22 (4) | 531 (100) | 0 (0) | 4 (44) | 5 (56) | 9 (100) |  |  |  |  |
| **Gravidity** |  |  |  |  |  |  |  |  |  |  |  |  |
| First pregnancy | 1586 (63) | 240 (10) | 694 (27) | 2520 (100) | 57 (3) | 999 (56) | 739 (41) | 1795 (100) |  |  |  |  |
| Not first pregnancy | 5560 (86) | 186 (3) | 711 (11) | 6457 (100) | 442 (10) | 2244 (52) | 1604 (38) | 4290 (100) |  |  |  |  |
| Missing |  |  |  | 1 (100) |  |  |  |  |  |  |  |  |
